# Wearable CNT/PAni/fabric piezoresistive sensor for continuous blood pressure monitoring

**DOI:** 10.1017/wtc.2024.31

**Published:** 2025-02-10

**Authors:** Milad Barati, Alireza Nikfarjam

**Affiliations:** MEMS & NEMS Lab, Department of Microsystem and Photonics, School of Intelligent Systems Engineering, University of Tehran, Tehran, Iran

**Keywords:** micro pressure sensor, fabric, CNTs, PAni, pulse transit time, blood pressure

## Abstract

Wearable pressure sensors with high sensitivity, fast response time, and low detection limit have great potential for blood pressure monitoring and early diagnosis of hypertension. This article introduces a piezoresistive pressure sensor based on carbon nanotubes (CNTs), polyaniline (PAni), and fabric (CNT/PAni/fabric) for health monitoring applications. This sensor is made by using two layers of linen fabric coated with CNT and PAni. These layers are placed on a polyester fabric substrate. One of the coated layers has a mesh structure, which increases the sensitivity of the sensor and lowers its detection limit. The CNT/PAni/fabric sensor has a high sensitivity of 2.035 kPa^−1^ at pressures from 0 to 0.2 kPa, a response time of 290 ms, and a detection limit of 1.5 Pa. These features make it suitable for measuring blood pressure. The results obtained by measuring blood pressure using the pulse transit time method on four people, compared with the values obtained using the digital sphygmomanometer, show a discrepancy ranging between 0.019% and 1.62%. Also, the average error and standard deviation for the sensor measurement in systolic and diastolic pressures are 0.56 ± 0.33 and 0.57 ± 0.46, respectively, which shows that measurement with this sensor can be an alternative to existing devices.

## 1. Introduction

Pressure sensors have a wide range of applications in soft robotic skin (Zhan et al., 2017), human motions detection (Ge et al., 2018), energy harvesting (Cheng et al., 2018), medical diagnostics (Li et al., 2020), and health monitoring (Huang et al., 2019). The key features of these sensors are high sensitivity, low weight, and low-cost fabrication processes. These characteristics demonstrate the high potential of pressure sensors in health monitoring. Blood pressure measurement is one of the major challenges in this field. High blood pressure is a major risk factor for cardiovascular disease, including heart attack, aneurysm, and heart failure. High blood pressure can be controlled by lifestyle changes and medication, but because it has no symptoms, it often goes unnoticed by most people. Therefore, there is a need for a wearable system that can continuously measure blood pressure (Kim et al., 2020; Kumar, 2021).

Pressure sensors are devices that convert mechanical pressure into electrical signals. Parameters such as linearity, sensitivity, detection limit, response time, and stability are used to describe their performance. The operating mechanism of these sensors can be piezoresistive (Cao et al., 2021), triboelectric (Chen et al., 2020), piezoelectric (Sreeja and Sankararajan, 2022), and piezocapacitive (Kwon et al., 2016). However, piezoresistive and piezocapacitive sensors are the most promising for wearable applications. The piezocapacitance mechanism can be thought of as a parallel plate capacitor. When pressure is applied, the distance between the two plates decreases and the capacitance increases. In this example of these sensors, two parallel silicon plates, on which silver nanowires are placed, are used as capacitor plates. The lines of silver nanowires (AgNWs) are 2 mm long and placed 2 mm apart in the silicon plate, and their ends are made of gallium indium liquid metal for bonding. The lines of AgNWs are superimposed on two planes with an angle difference of 90°. Ecoflex is used as the dielectric layer between the two plates. The sensitivity for pressures below and above 500 kPa is 1.62 Mpa^−1^ and 0.57 Mpa^−1^, respectively. The response time and detection limit are 40 ms and 0.6 g, respectively (Yao and Zhu, 2014). Piezoresistive sensors are devices that change their electrical resistance in response to pressure. Electrical resistance indicates how well a device reduces current. This response is typically caused by a change in contact resistance between two materials or surfaces due to mechanical deflection caused by pressure. Gao et al. (2018) conducted research by casting PDMS on sandpaper and using it as a mould. They then used the formed PDMS as a mould and added a mixture of PDMS and carbon nanotubes (CNTs). Finally, they placed two samples of the same structure face-to-face to create a low-cost sensor. This sensor has a sensitivity of 0.2 kPa^−1^, a detection limit of 5 Pa, and a response time of 190 ms. When comparing piezoresistive and piezocapacitive mechanisms, capacitive pressure sensors are highly sensitive to static deformation. In contrast, piezoresistive sensors perform well in detecting transient or dynamic deformation due to the intermittent nature of their piezoelectric effects. Piezoresistive mechanisms also offer advantages such as easy fabrication methods, simple readout mechanisms, fast response times, and easy signal acquisition. Another feature of piezoresistive sensors is their deficient power consumption, making them more popular in recent years (Ding et al., 2019; Cao et al., 2021).

To improve the performance of piezoresistive sensors, various materials have been used in their structure to reduce their initial resistance. Conductive polymers such as polypyrrole (Wang et al., 2016), PEDOT: PSS (Ding et al., 2018), or polyaniline (PAni; Chen et al., 2022) have proven to be superior alternatives to other expensive conductive materials (Kannichankandy et al., 2021). PAni can be an excellent alternative due to its favorable properties. It has an inexpensive monomer and a convenient synthesis method. In addition, features such as high thermal, electrical and chemical stability, environmental compatibility, and the ability to protect against corrosion are among its advantages (Visakh et al., 2017; Mozafari and Chauhan, 2019). Zheng et al. (2019) reported microcracked and cavitated PAni foams supported by polydimethylsiloxane (PDMS), which showed a sensitivity of 0.055 kPa^−1^ in the range of 0–5 kPa and its detection limit was 4 Pa. PAni is combined with other materials in newer technologies due to its fragile structure, medium conductivity, and short charge/discharge cycle life. Carbon materials such as graphene (Huang et al., 2019), reduced graphene oxide (rGo; Zheng et al., 2020), and CNTs (Hong et al., 2017) are ideal candidates for integration with PAni due to their exceptional electron mobility, mechanical stability, and thermal stability. The exceptional mechanical properties of CNTs, including their high Young’s modulus and tensile strength, make them optimal and promising reinforcements for significantly improving the mechanical properties of polymer–CNT composites (Arash et al., 2014). The addition of CNTs to PAni not only significantly increases its electrical conductivity but also improves its stability, effective surface area, and brittleness (Zhang et al., 2008). Xiao et al. (2023) prepared a 3D conductive foam from polyurethane, CNT, and PAni. This foam showed impressive electrical conductivity and durability, resulting in a significant response over a wide range of 0–30,000 kPa.

Here, we have demonstrated a sensitive piezoresistive sensor based on a mesh structure of linen fabric coated with PAni and CNT. The sensor consists of two linen fabrics coated on a polyester fabric substrate. One of the linen fabrics has a mesh structure which increases the air space between the two layers, increasing the sensitivity of the sensor and reducing its detection limit. The two-layer PAni/CNT/fabric sensor has a sensitivity of (2.035 kPa^−1^) in the range (0–200 Pa) and a detection limit of (1.5 Pa), making it suitable for measuring pulse waves and blood pressure. Considering the high risk of hypertension disease and the importance of its prevention, it seems necessary to develop a blood pressure monitoring system that can be used to measure blood pressure at home without the need for a monitor.

## 2. Experimental section

### 2.1. Fabrication of CNT/PAni/fabric layer and pressure sensor

**Figure 1.**
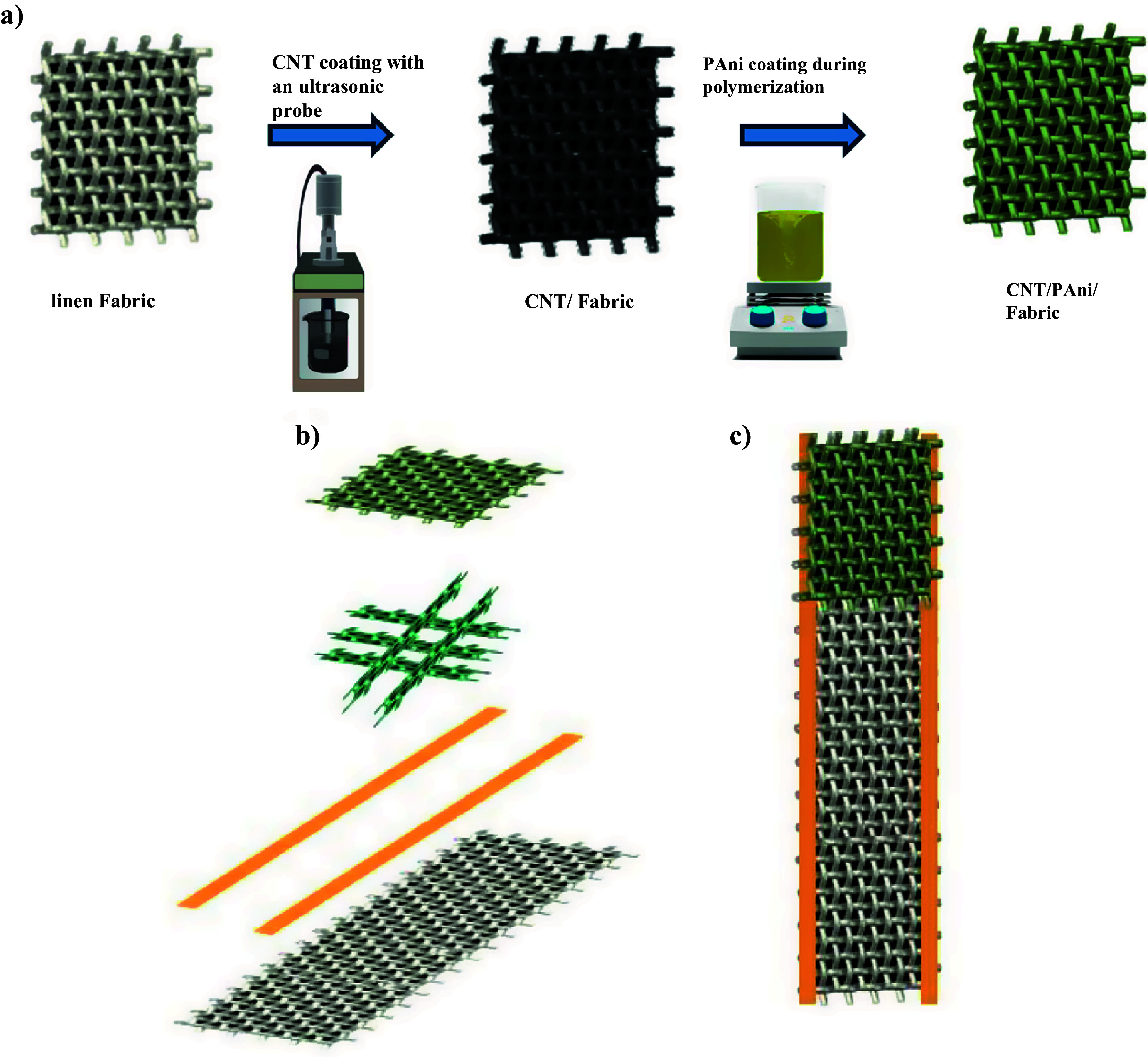
Fabrication process of CNT/PAni/fabric. (a) Overview of the process to fabricate the sensing layer (CNT/PAni/fabric). (b) Different parts of the sensor. (c) The final shape of the sensor.

The fabrication process of the CNT/PAni/fabric layer is shown in Figure 1(a). Linen fabrics are cut into 1 × 1 cm^2^ pieces. Before coating, they are washed three times with deionized water (DIW) and ethanol. The fabric is washed in an ultrasonic bath. The washing time is set to 480 s. To prepare the CNT solution, 80 mL of DIW is added to 28.4 mg of CNT. The linen fabric is immersed in the solution and an ultrasonic probe is used to distribute the CNT evenly on the fabric. The mixture is stirred several times for 10 min each, to ensure complete coverage of the canvas. Then the fabrics from the solution are removed and allowed to dry completely in an oven for 10 min.

The next step is to make two separate solutions, A and B, to create a PAni solution. Solution A is made by combining 60 mL of DIW with 4.8 mL of hydrochloric acid (HCl). After stirring for 15 min, 4 mL of aniline is added and the solution is placed at 0°C for polymerization. Solution B is prepared by combining 24 mL of DIW with 1.95 mL of HCl and then adding 0.7 g of ammonium persulfate (APS). Similar to Solution A, it is stirred for 15 min and placed at 0°C. The coated fabrics are immersed in Solution A, followed by the dropwise addition of Solution B. To complete the polymerization process, the resulting solution is kept at 0°C for 20 hr. The tissues are then removed from the solution and washed several times with DIW.

To make the sensor substrate, the polyester fabric is cut into 5 × 2 cm^2^ pieces and then washed in an ultrasonic bath for 480 s, first with DIW and then with ethanol. This process is repeated several times to ensure the cleanliness of the fabric. To dry, the fabric is placed in an oven at 80°C for 10 min. Once the polyester fabric is dry, the double-sided copper tape is cut to a size of 1 × 5 cm^2^ and placed vertically on both sides of the polyester fabric. The copper tape plays the role of the electrode in two layers and the entire sensor output, which is connected to the polyester fabric. To make the final sensor, the pre-prepared linen fabric coated with PAni and CNT is divided into smaller dimensions of 0.3 × 1 cm^2^. Five pieces of fabric are selected and placed on the polyester fabric. Three pieces of coated fabric are placed horizontally on top of the sensor, between two copper strips, at a distance of 5 mm. The remaining two pieces of fabric are placed vertically on top of these three pieces. Next, another 1 × 1 cm^2^ piece of linen fabric coated with PAni and CNT is placed on these parts. Finally, the sensor is encapsulated on both sides with masking tape.

### 2.2. Characterization of CNT/PAni/fabric layer and measurements

An electron microscope is used to observe the morphology of the sample surface using scanning electron microscopy (SEM) and energy-dispersive X-ray spectroscopy (EDS) analyses. The SEM analysis was performed at 20 kV voltage and the EDS measurement was also performed with energy between 0 and13 keV. CNT and PAni coating on linen fabric can also be confirmed by Fourier transform infrared spectroscopy (FTIR) analysis. For FTIR, an instrument (Perkin Elmer spectrometer) was used and the wavelength between 400 and 4,000 cm^−1^ was analyzed.

A digital multimeter (Victor VC97) was used to measure the resistance of the sensor. A digital oscilloscope (RIGOL DS 1054) was used to measure the voltage changes due to the application of pressure and a voltage source (PS-405 U2) was used to supply the input voltage. A resistive divider circuit is used to convert changes in sensor resistance due to pressure into changes in voltage. To have a digital output of the circuit and finally to display the voltage changes resulting from pressure changes as changes in blood pressure, an Arduino board (Arduino UNO R3) is used and the output signal is connected to one of the analog-to-digital conversion pins.

To calibrate the sensors, it is necessary to use a device that can measure blood pressure in real time. After recording the signals from two sensors using the Arduino board and its digital display, the final step is to use a cuff-based sphygmomanometer (Omron M6 Comfort) to obtain the people’s actual blood pressure.

## 3. Results and discussion

### 3.1. Pressure sensor performance

**Figure 2.**
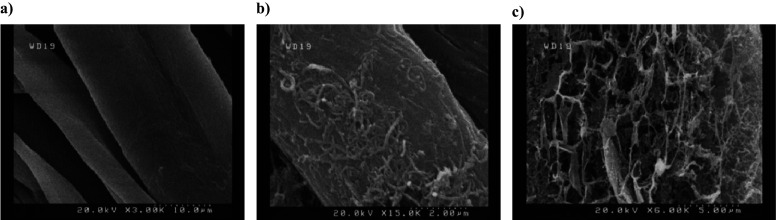
SEM images: (a) Linen Fabric at 3,000x magnification, (b) CNT/fabric at 15,000x magnification, and (c) CNT/PAni/fabric at 6,000x magnification.

First, the linen fabric was coated with CNT solution to create the pressure-sensitive layer. The CNT-coated fabric was then immersed in the PAni solution during polymerization. To fabricate the sensor, a mesh-like CNT/PAni/fabric structure is placed on polyester and covered with a 1 × 1 cm^2^ square fabric, as shown in Figure 1. The detailed fabrication process of the designed device is given in the experimental section. The CNT and PAni coating on the linen fabric was identified by SEM images, as shown in Figure 2. Figure 2(a) shows the SEM image of the linen fabric before coating. The fibers of this fabric are irregularly distributed, creating a large air gap between them. The surface of the uncoated fabric is completely smooth and the average length of each fiber is 150–200 μm. After CNTs are added to the fabric, a curved tubular structure appears on its surface (Figure 2(b)). Figure 2(c) shows that PAni forms a cluster-like network of nanowires on the surface of the linen fabric. This dense network of PAni fibers increases the roughness and electrical conductivity of the fabric. Table 1 shows the percentage by weight of the elements in the CNT/PAni/fabric based on EDS analysis and also gives the percentage by mass of each element. The chemical structure of linen fabric is similar to that of cellulose cotton and the presence of cellulose ((C_6_H_10_O_5_)*_n_*) has caused the presence of carbon and oxygen in the EDS analysis. Also, after the addition of CNT, the percentage of carbon in the structure has increased compared to other elements. According to Table 1, there has been a definite absorption of PAni on the fabric, which can be detected by the presence of elements such as nitrogen, sulfur, and chlorine. Nitrogen and sulfur are related to the presence of APS ((NH_4_)_2_S_2_O_8_) in the formation of PAni and Cl is formed by the addition of HCl (Muthukumar et al., 2016).

**Table 1.** Weight percentage of elements in CNT/PAni/fabric based on EDS analysis

Number	Elements	Weight percent (%)
1	Carbon (C)	54.3%
2	Nitrogen (N)	1.5%
3	Oxygen (O)	11.8%
4	Sulfur (S)	14.5%
5	Chlorine (Cl)	17.9%
	All	100%

**Figure 3.**
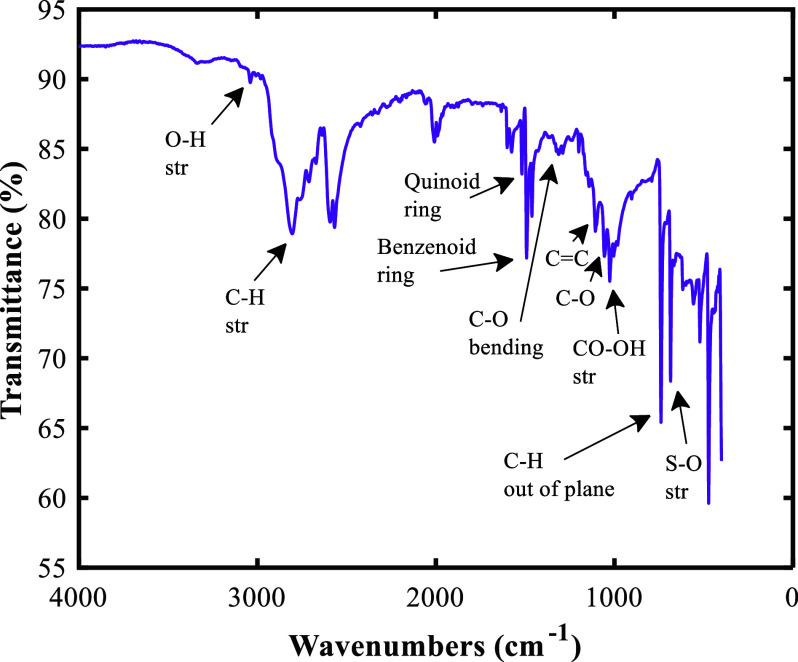
FTIR analysis for CNT/PAni/fabric.

Figure 3 shows the results of the FTIR analysis for CNT/PAni/fabric. The long peak at wave number 3039.5 cm^−1^ is the characteristic peak of the hydroxyl groups (OH) of cellulose. A peak at 2,804.5 cm^−1^ is seen, which is related to the stretching vibration of the C-H bond in cellulose. The absorption at 1312.8 cm^−1^ is related to the bending vibration of the C-O group, which is related to the aromatic ring of the polysaccharide. The strong peak observed at 1,019.19 cm^−1^ is related to the stretching vibration of CO with OH in cellulose polysaccharides (Chung et al., 2004; Portella et al., 2016). The peak in the range of 1,100–1,200 cm^−1^ (1,101.6 cm^−1^) confirms the hexagonal structure of CNT, which is related to the C=C bond of the CNT skeletal vibration mode. The peak at 1,054 cm^−1^ is also related to the C-O vibration of the ester, phenol, or carboxyl group, which makes it difficult to give a definite opinion due to overlap (Lin et al., 2008; Ţucureanu et al., 2016). The two peaks at 1,575 cm^−1^ and 1,493.3 cm^−1^ are related to the stretching vibration of the quinoid ring and the benzenoid ring. The peak at 740.59 cm^−1^ characterizes the out-of-plane C-H bond in the aromatic ring and the stretching vibration of the S-O bond of the sulfonate groups attached to the aromatic ring causes the absorption at 682.69 cm^−1^ (Shao et al., 2012; Sharma and Sharma, 2013).

The sensor mechanism is based on resistance changes caused by pressure changes. When pressure is applied to the sensor, the fabric on top makes more contact with the linen fabric below. This contact increases the conductivity between the two copper electrodes. In addition, applying pressure reduces the amount of air trapped between the mesh and the upper fabric, thereby reducing the resistance. When the pressure is released, the sensor returns to its original state and resistance level.

**Figure 4.**
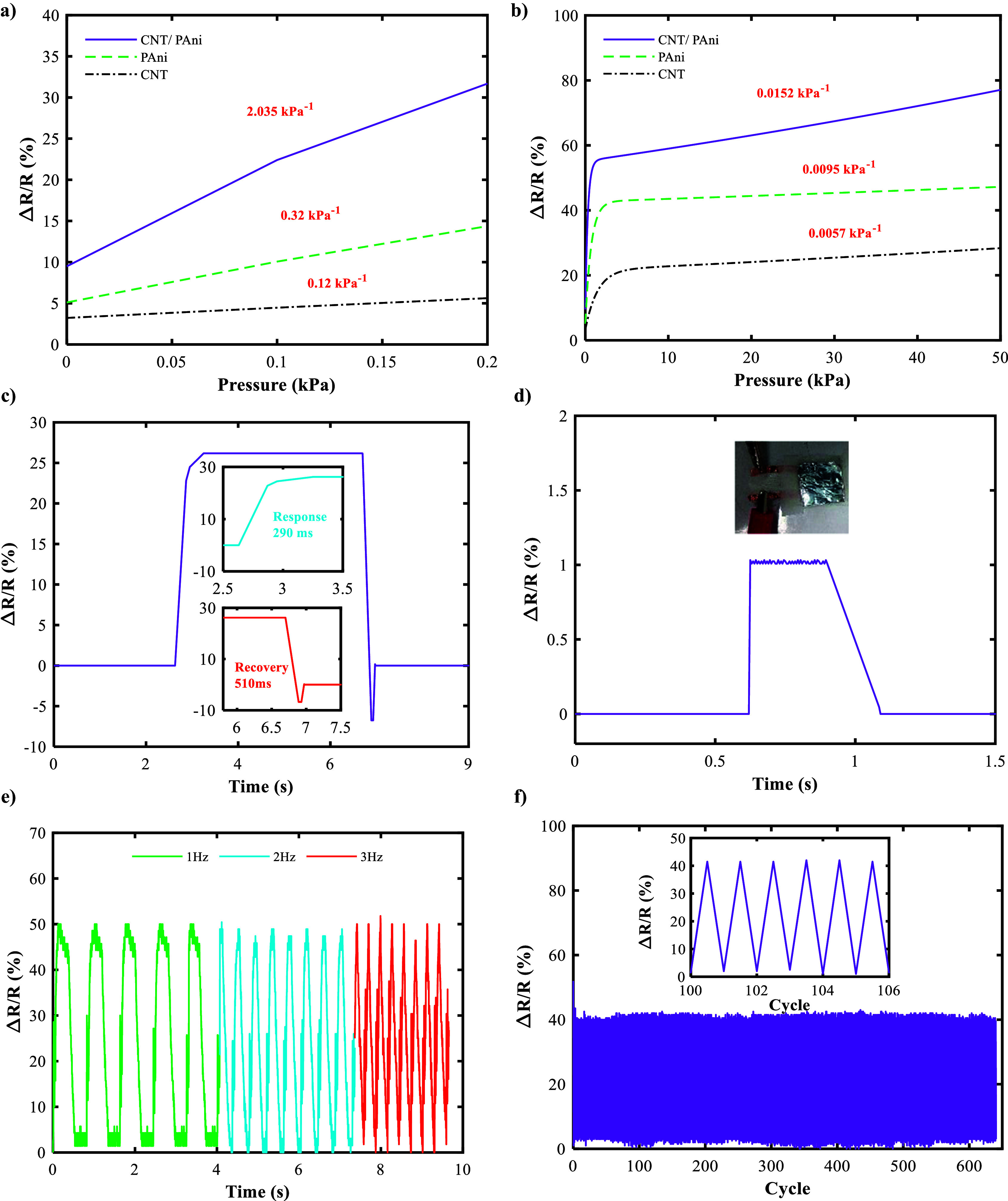
Diagram of changes in resistance (sensitivity) of sensors made of a combination of fabric with CNT, PAni, and a combination of CNT/PAni by applying a pressure of (a) 0–0.2 kPa and (b) 0–50 kPa. (c) Response time of CNT/PAni/fabric sensor by applying pressure of 0.1 kPa. (d) Detection limit of CNT/PAni/fabric sensor by applying 1.5 Pa pressure. (e) CNT/PAni/fabric sensor response by applying 1 kPa pressure at 1 Hz, 2 Hz, and 3 Hz frequencies. (f) CNT/PAni/fabric sensor response by applying a constant pressure of 1 kPa for 650 cycles.

The combination of CNT and PAni on fabric results in a highly sensitive sensor that operates in two linear ranges. The sensitivity of the sensor can be calculated using Equation (1):(1)

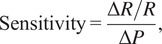

where Δ*R* and *R* are the changes in resistance and initial resistance respectively, and Δ*P* is the applied pressure. The sensitivity of the sensor is shown in Figure 4. Figure 4 shows that in the low-pressure range (0–0.2 kPa), the sensitivity has increased much faster. The rapid increase in conductive pathways between the two electrodes is the primary reason for this effect. The current flow from one electrode to the other electrode on both sides of the polyester fabric is mostly through the lower fabric, which has a mesh-like structure, which has empty holes in its structure. In pressures <0.2 kPa, these holes are gradually filled by the upper fabric, creating a noticeable change in the overall resistance of the path. By increasing the pressure to 0.2 kPa, almost all of the empty holes in the mesh-like structure of the lower fabric are filled by the upper fabric, and the two surfaces are completely attached and applying more pressure does not change the overall resistance of the path, and the sensitivity is noticeably reduced. The reason for the decrease in sensitivity is also the saturation of the number of conductive paths between the two copper electrodes (Luo et al., 2017; Chen et al., 2018; Gao et al., 2018; Park et al., 2018; Xia et al., 2018; He et al., 2019). This pressure range is critical for blood pressure measurement. Figure 4(a) shows a sensitivity diagram for three different sensors. The CNT/PAni/fabric sensor has a sensitivity of 2.035 kPa^−1^, which is much better than the PAni/fabric (0.32 kPa^−1^) and CNT/fabric (0.12 kPa^−1^) sensors. This indicates that the combination of these two materials has been very effective in achieving high sensitivity at low pressures. Figure 4(b) shows the measuring range of the sensor, which is 0–50 kPa, together with the sensitivity for the whole range. The CNT/PAni/Fabric sensor has a higher sensitivity than the PAni/Fabric and CNT/Fabric sensors over the entire range. The sensitivities of the CNT/PAni/fabric, PAni/fabric, and CNT/fabric sensors are 0.0152 kPa^−1^, 0.0095 kPa^−1^, and 0.0057 kPa^−1^ respectively.

To calculate the response time of the CNT/PAni/fabric sensor, the resistance changes of the sensor were measured by applying a certain pressure. According to Figure 4(c), by applying a pressure of 0.1 kPa, the response time and recovery time of the sensor were 290 ms and 510 ms, respectively.

The detection limit was obtained by placing a piece of aluminium sheet measuring 1.5 × 2 cm^2^ and weighing 45 mg, which applied a pressure of 1.5 Pa to the sensor (Figure 4(d)).

Figure 4(e) shows the changes in resistance of the fabric/CNT/PAni sensor at frequencies of 1 Hz, 2 Hz, and 3 Hz. In particular, when the sensor is subjected to the same pressure of 1 kPa, the output signal curve remains constant. This observation suggests that the response of the sensor to constant pressure is independent of frequency. At a frequency of 1 Hz, the initial range of the sensor showed a change of 48.6%. This range remains constant at 1 Hz, while at frequencies of 2–3 Hz, the maximum deviation from the initial range is 4.32%, which is within an acceptable range. Figure 4(f) shows the output of the sensor when a constant pressure of 1 kPa is applied for 650 cycles. The cycle ranges show a maximum percentage difference of 1.5%.

### 3.2. Application: blood pressure monitoring system

**Figure 5.**
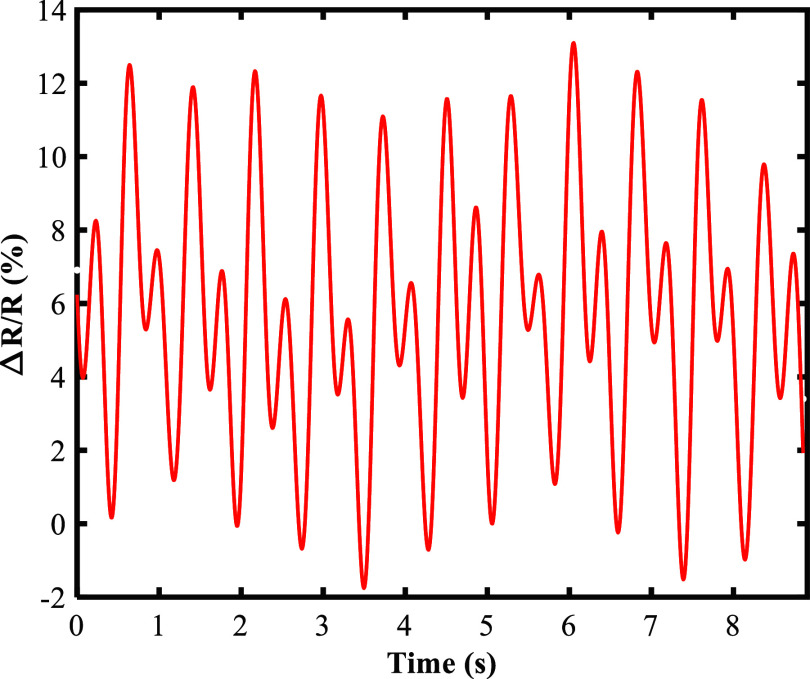
Waveform of the pulse signal obtained from the CNT/PAni/fabric sensor on the neck.

To accurately measure blood pressure, the sensor must be highly sensitive and have a very low detection limit for pulse pressure measurement. In addition, the response time of the sensor is crucial for this measurement. Since the interval between two heartbeats is ~900 ms, the sensor must have a total response and recovery time of <900 ms to effectively detect a heartbeat. A CNT/PAni/fabric sensor with a total response and recovery time of 800 ms and a detection limit of 1.5 Pa is a suitable sensor for blood pressure measurement. This sensor can measure the smallest dynamic information of changes in the arteries of the human body, to monitor the pulse signal and then calculate the blood pressure. As shown in Figure 5, due to the high sensitivity of the sensor to external pressure changes, all characteristic points of the pulse waveform are measured correctly. The CNT/PAni/fabric sensor can only detect mechanical changes associated with blood pressure. A measurement kit is required to measure blood pressure. This set consists of a sensor to receive a mechanical signal, a power supply for the sensor, a circuit to manage and process the signal, and a system to display the output signal. All the components in this set work together to provide continuous blood pressure monitoring. The average heart rate for an individual is around 1 Hz, so the circuitry has been optimized to filter out signals at other frequencies. The signal is then sent to the Arduino board and its analog-to-digital conversion (ADC) section. The ADC operates at a sampling rate of 15k samples/s. The highly accurate 10-bit analog-to-digital converter and the appropriate sampling rate ensure that the information is stored in great detail. The microcontroller unit (MCU) on the Arduino board is responsible for processing the data. Once the information has been processed, the results are transmitted.

To calculate blood pressure using the Mons–Korteweg formula, it is important to determine the time difference between the blood reaching two different points in the body, assuming that the elastic modulus of the vessel walls remains constant. In humans, the elastic modulus of the vessel walls does not change significantly over short periods of time, so the pulse transit time (PTT) is closely related to blood pressure and is influenced by physiological characteristics. Therefore, Equation (2), derived from the Mons–Korteweg formula, can be used to calculate blood pressure in humans (Chen et al., 2000; Shriram et al., 2010).(2)

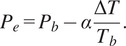



In Equation (2), Δ*T* refers to the change in time taken for the pulse to travel from Point A to Point B. Tb represents the amount of PTT related to the Pb pressure. In addition, Pe represents the secondary pressure. The α coefficient is also a unique value determined by calibration and is different for each person.

For blood pressure monitoring, the functionality of the sensor was tested on healthy subjects according to standard criteria. First, the stability of the sensor was assessed on three individuals. Subsequently, five different sensors were used on a single individual to assess the reproducibility of sensor manufacture and to ensure consistent results. To demonstrate the ability of the sensor to monitor pressure, two sensors were placed on the subject’s wrist and neck, where the radial artery passes and the received signal is stronger. The subject was then asked to hold the palm of their hand up and stretch a little more than usual to make the radial artery more visible on the surface of the skin. During the measurement, the person held the pressure sensor close to their heart. No allergic reaction or pain was reported by the subject. Adhesive tape is used to attach the sensor to the skin of the hand, and the pressure on the sensor when placed on the skin is high to ensure good contact with the skin. This pressure reduces the initial resistance of the sensor. As blood pressure increases, the radial artery expands and the surrounding tissue deforms, causing the sensor to deform and change its resistance. The transit time is obtained by measuring the change between two pulses.

The blood pressure is first measured by an electronic sphygmomanometer and then the PTT is obtained directly from the sensors. Important components in measuring blood pressure are systolic pressure and diastolic pressure. These are among the most commonly tested components when assessing a person’s cardiovascular health.

To obtain the most accurate results, three consecutive blood pressure readings were taken with the electronic sphygmomanometer and the fourth reading was used to calibrate the sensor.

**Figure 6.**
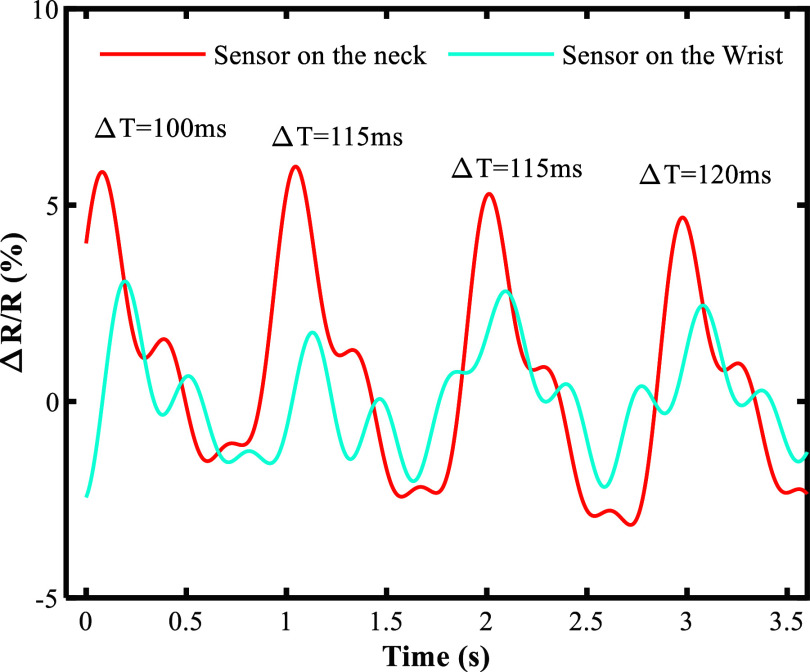
Two waveforms were received from two sensors, and the time difference between two peaks in the signals was calculated for a blood pressure reading of 131/65 mmHg.

Figure 6 shows a diagram of the sensors on the subject. The time difference between the two pulse signals reaching the sensors was between 90 and 120 ms. To ensure accurate readings, the signals were averaged over four cardiac cycles and compared with an electronic sphygmomanometer. In this case, the average PTT was 112.5 ms. According to Equation (2), the systolic and diastolic blood pressures obtained by the sensor were 131.96 and 64.98, respectively. The systolic and diastolic blood pressures were measured using an OMRON cuff blood pressure monitor, which indicated 131 mmHg and 65 mmHg, respectively. The measurement error in this case is 0.7% for systolic pressure and 0.019% for diastolic pressure. To demonstrate the feasibility of the wearable CNT/PAni/fabric sensor system for noninvasive continuous biomedical monitoring, we also performed cardiovascular parameter (BP) measurements on three additional subjects. These subjects ranged in age from 26 to 34 years and had different health conditions. For each participant, we first used our sensor system to measure their blood pressure. Then, to validate the accuracy of the BP measurements, we used a cuff-based sphygmomanometer (OMRON) to assess the BP of the same individual. A statistical result of the systolic and diastolic BP measurements is shown in Table 2.

**Table 2.** Results of systolic and diastolic blood pressure measurement for four participants

	Subject A	Subject B	Subject C	Subject D
SBP by sensor	131.96	125.12	130.83	127.33
DBP by sensor	64.98	78.75	70.83	74.13
SBP by cuff-based device	131	125	130	127
DBP by cuff-based device	65	79	72	75
SBP measurement discrepancy	0.7%	0.1%	1.15%	0.44%
DBP measurement discrepancy	0.019%	0.31%	1.62%	1.16%

*Note.* SBP monitoring discrepancy = |SBPWCSPS − SBPcuff-based|/SBPcuff-based × 100%; DBP monitoring discrepancy = |DBPWCSPS − DBPcuff-based|/DBPcuff-based × 100%).

Among the persons tested, Subject C has high blood pressure, and according to Table 2, he has the highest discrepancy in general, a low discrepancy (between 0.019% and 1.62%) between the measured blood pressure and the results obtained from the sensor shows that this sensor can be used as a device for blood pressure measurement.

The average measurement error of the sensor and the standard deviation were also calculated and their value for systolic and diastolic pressure were 0.056 ± 0.33 mmHg and 0.057 ± 0.46 mmHg, respectively. According to ISO 81060-2, noninvasive blood pressure can be replaced by an arterial catheter if the mean distortion is <5 mmHg and the standard deviation is <8 mmHg according to AAMI (Kim et al., 2019).

## 4. Conclusion

In this study, we have developed a wearable piezoresistive sensor using CNT and PAni as the pressure-sensitive layer. This sensor has a wide measurement range of 0–50 kPa and a high sensitivity of 2.035 kPa^−1^ within the range of 0–0.2 kPa. It has a fast response time of 290 ms, a recovery time of 510 ms, and an extremely low detection limit of 1.5 Pa. The pressure sensor has also demonstrated good stability over time and with the application of specific pressure at different frequencies, confirming the sensor’s capability.

We have also demonstrated that this sensor can be used as a blood pressure measurement system. By placing two CNT/PAni/fabric sensors on the wrist and neck of the subjects, where the strongest pulses were received from these points, we received the pulse signal well. Then, using the PTT method and data transmission, we were able to measure the blood pressure using Arduino and display the output on the laptop‥ The blood pressure values obtained using the CNT/PAni/Fabric sensor were very close compared to the blood pressure values shown on the digital sphygmomanometer, indicating the effectiveness of this sensor for measuring blood pressure. One of the advantages of this system is the continuous measurement of blood pressure and its portability, which promises to be part of the new generation of blood pressure measurement systems. Given the asymptomatic nature of a disease such as hypertension, a system capable of continuously measuring blood pressure can be very useful in its prevention.

## Data Availability

Data sets generated during the current study are available from the corresponding author on reasonable request.
